# A Cross-Sectional Analysis of Speaker Representation: Intentionality meets Intersectionality in Academic Global Surgery

**DOI:** 10.5334/aogh.4972

**Published:** 2025-11-07

**Authors:** Brenda Feres, Eilene Basu, Anna Mary Jose, Rabbey Raza Khan, Abeba Aleka Kebede, Camila Sotomayor, Marina Reis, Kaela Blake, Jessica L. Buicko Lopez, Tanaz Vaghaiwalla

**Affiliations:** 1 Gender Equity Initiative in Global Surgery, Boston, MA, USA; 2 Kursk State Medical University, Kursk, Kurskaya Oblast, Russia; 3 Datta Meghe Institute of Medical Sciences, Wardha, India; 4 Bolan University of Medical and Health Sciences, Quetta, Pakistan; 5 Pontifical Catholic University of Chile, Santiago, Chile; 6 Federal University of Health Sciences of Porto Alegre, Porto Alegre, Brazil; 7 Department of Surgery, University of Tennessee Medical Center, Knoxville, TN, USA; 8 University of Texas Health Science Center at Houston, Department of Surgery, Houston, TX, USA; 9 Division of Endocrine Surgery, DeWitt Daughtry Family Department of Surgery, University of Miami Leonard M. Miller School of Medicine, Miami, FL, USA

**Keywords:** global surgery, gender equity, academic surgery, LMICs

## Abstract

*Introduction:* Despite increased advocacy, women and professionals from low- and middle-income countries (LMICs) remain underrepresented in global surgery. To address this, the Gender Equity Initiative in Global Surgery (GEIGS) launched an annual General Assembly (GA). This study evaluates trends in academic representation and leadership at the GA, emphasizing pathways to equitable mentorship, academic voice, and faculty development.

*Methods:* A retrospective cross-sectional analysis of GEIGS GA speakers from 2020 to 2025 was performed. Data from conference records and public sources included gender, country of practice, degree(s), leadership role, citation count, prior speaking experience, and topic area. Linear regression assessed trends in gender representation.

*Results:* A total of 104 speakers were identified. Women comprised 83.7% (*n* = 87; *p* < 0.001). Leadership positions were held by 64.4% (*n* = 67). The most common degrees were MD (62.5%) and PhD (16.4%); 15.4% (*n* = 16) were medical students. Of the speakers, 60.6% (*n* = 63) practiced in high-income countries (HICs) and 39.4% (*n* = 41) in LMICs. Representation by women was consistent across regions: 84.1% in HICs and 82.9% in LMICs. Among LMIC speakers, 65.9% held leadership roles, 34.2% multiple degrees, and 68.3% prior speaking experience. No significant differences in academic qualifications were observed between HIC and LMIC speakers (*p* > 0.05).

*Conclusion:* The GEIGS GA demonstrates that intentional, equity-focused planning can achieve high female participation and meaningful inclusion of LMIC professionals. These findings provide a practical framework for promoting equitable representation in global surgery conferences. Continued attention to supporting LMIC voices in leadership and academia may help sustain progress.

## Introduction

Global surgery has emerged as a critical academic and clinical field aimed at strengthening health systems worldwide and addressing disparities in access to surgical care, particularly in underserved regions [1–3]. Achieving universal health coverage and equitable surgical access requires strategies that integrate advocacy, capacity building, sustainable interventions, and gender-inclusive frameworks within surgical systems [4]. Gender equity in leadership and representation is essential to ensure that policies and practices reflect the diverse populations they are intended to serve [5].

Despite growing advocacy, women and professionals from low- and middle-income countries (LMICs) remain underrepresented in the academic discourse on global surgery [6]. Initiatives such as the Gender Equity Initiative in Global Surgery (GEIGS) were developed to promote equity through deliberate inclusion of underrepresented groups, including consideration of gender, geography, and professional status [7, 8]. The GEIGS General Assembly (GA) is an annual international meeting conducted virtually with a focus on amplifying the voices of women and professionals from LMICs [9].

This study examines speaker representation at the GEIGS GAs from 2020 to 2025, with attention to gender, geographic distribution, academic credentials, leadership roles, and specialty representation. By evaluating trends in representation and academic involvement, the study aims to identify strategies that may promote equitable participation and provide a framework for mentorship, leadership development, and inclusive program design in global surgery.

## Materials and Methods

### Study design and setting

A cross-sectional study was conducted to analyze data from all invited guest speakers at the GEIGS GA from 2020 to 2025. The 2022 GA was excluded because the event was not held that year. This study was reviewed and approved by the University of Tennessee Medical Center Institutional Review Board, and the requirement for informed consent was waived because the study involved minimal risk and used de-identified data.

### Data collection

Data were retrospectively collected from GEIGS records and publicly available sources. Variables included gender, specialty, country of practice, highest degree earned, number of citations, prior conference experience, and presentation topics. Gender was self-reported and extracted from anonymized GA data. Countries were classified as high-income or low/middle-income based on the 2024–2025 World Bank classification [10].

### Statistical analysis

Descriptive statistics summarized variables across the five assemblies. Categorical variables were expressed as frequencies and percentages, and continuous variables as medians with interquartile ranges. Linear regression analyzed gender representation and credential differences between HIC and LMIC speakers. Mann–Whitney *U* tests compared citation counts between the two groups. Statistical significance was set at *p* < 0.05. Data were organized in Microsoft Excel and analyzed using STATA (version 18.0).

## Results

During the study period, 104 speakers participated in the GEIGS GA. In 2020, there were a total 22 speakers (21.2%), followed by 19 speakers (18.3%) in 2021, 21 speakers (20.2%) in 2023, 24 speakers (23.1%) in 2024, and 18 speakers (17.3%) in 2025. Overall, females comprised 83.7% (*n* = 87/104) of the speakers (*p* < 0.001; β = 14.0; 95% CI [10.49–17.41]). In 2020, 86.4% (*n* = 19/22) of the speakers were women, followed by 73.7% (*n* = 14/19) in 2021, 90.5% (*n* = 19/21) in 2023, 87.5% (*n* = 21/24) in 2024, and 77.8% (*n* = 14/18) in 2025.

### Speakers’ credentials

Overall, speakers holding a leadership position comprised 64.4% (*n* = 67/104). Speakers with multiple degrees were 36.5% (*n* = 38/104). The most common academic degree was an MD (*n* = 65/104, 62.5%), followed by PhD (*n* = 17/104, 16.4%) and masters’ degree (*n* = 5/104, 4.8%). Medical students accounted for 15.4% (*n* = 16/104). The speakers who had previous experience presenting at other events were 73.1% (*n* = 76/104). The overall median of speakers’ research citation numbers was 116 with the interquartile range [0–599] (Table 1).

**Table 1:** Speakers’ characteristics.

	GENERAL ASSEMBLY YEAR					
	2020	2021	2023	2024	2025	Total
Number of speakers *n*, (%)	22 (21.2)	19 (18.3)	21 (20.2)	24 (23.1)	18 (17.3)	104
Female speakers, *n* (%)	19 (86.4)	14 (73.7)	19 (90.5)	21 (87.5)	18 (17.3)	87 (83.7)
Speakers with leadership positions, *n* (%)	14 (63.6)	9 (47.4)	11 (52.4)	20 (83.3)	13 (72.2)	67 (64.4)
Speakers with multiple degrees, *n* (%)	4 (18.8)	9 (47.4)	10 (47.6)	10 (41.7)	5 (27.8)	38 (36.5)
MD as a higher degree, *n* (%)	10 (45.4)	15 (78.9)	12 (57.1)	15 (62.5)	13 (72.2)	65 (62.5)
PhD as a higher degree, *n* (%)	1 (4.5)	4 (21.1)	5 (23.8)	5 (20.80)	2 (11.1)	17 (16.4)
Masters as a higher degree, *n* (%)	1 (4.5)	0 (0)	0 (0)	3 (12.5)	1 (5.6)	5 (4.8)
Medical students, *n* (%)	10 (45.4)	0 (0)	4 (19.0)	1 (4.2)	1 (5.6)	16 (15.4)
HIC speakers, *n* (%)	15 (68.2)	8 (42.1)	15 (71.4)	13 (54.2)	12 (66.7)	63 (60.6)
LMIC speakers, *n* (%)	7 (31.8)	11 (57.9)	6 (28.6)	11 (45.8)	6 (33.3)	41 (39.4)

*Note*: Table 1 describes the characteristics of the speakers across the five editions of the GEIGS General Assembly (2020–2025). HIC, high-income countries; LMIC, low- and middle-income countries.

### Speakers’ country of practice

**Figure 1:**
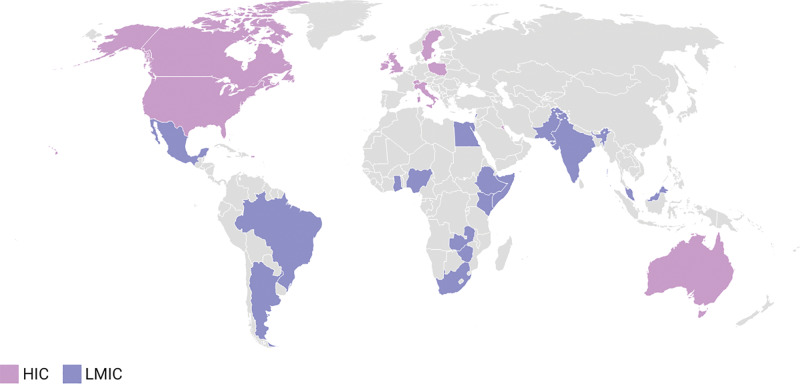
Speakers’ country of practice. ***Note*:** Figure 1 illustrates the geographic distribution of GEIGS General Assembly speakers by country, categorized by high-income countries (HICs) and low- and middle-income countries (LMICs). Created with Datawrapper.

The overall majority of speakers (*n* = 63/104, 60.6%) practiced in HICs, while 39.4% (*n* = 41/104) were from LMICs (*p* = 0.117; β = 4.4; 95% CI [–1.721, 10.521]) (Table 1). The countries representing HIC were the United Kingdom, United States, Switzerland, Sweden, Canada, Italy, Poland, Ireland, Kuwait, and Australia. The countries representing LMICs were Argentina, Brazil, Egypt, Ethiopia, Ghana, India, Kenya, Lebanon, Somalia, Malaysia, Mexico, Nigeria, Pakistan, Rwanda, South Africa, Zambia, Zimbabwe, and Venezuela (Figure 1).

Of the speakers practicing in LMICs, 82.9% (*n* = 34/41) were females, 65.9% (*n* = 27/41) had leadership positions, 34.1% (*n* = 14/41) possessed multiple academic degrees, and 68.3% (*n* = 28/41) had previously spoken at other events. The most common highest degrees were MD (*n* = 25/41, 61.0%) and PhD (*n* = 9/41, 22.0%). Medical students represented 12.2% (*n* = 5/41). The median number of research citations was 53 [0–410]. Regarding HIC speakers, 84.1% (n = 53/63) were women, 63.5% (n = 40/63) had leadership positions, 38.1% (n = 24/63) had multiple academic degrees, and 77.8% (n = 49/63) had previously spoken at other events. MD (n = 40/63, 63.5%) and PhD (n = 8/63, 12.7%) were the most common highest degrees. Speakers who were medical students comprised 17.5% (n = 11/63). The median of research citation numbers was 253 [45–753]. There were no significant differences in speaker credentials between HIC and LMIC (*p* > 0.05) (Table 2).

**Table 2:** Credentials of speakers in LMICs vs HICs.

	HIC (*N* = 63)	LMIC (*N* = 41)	*P*-VALUE	β; [95% CI]
Female speakers, *n* (%)	53 (84.1)	34 (82.9)	0.087	0.085; [–0.016 – 0.185]
Speakers with leadership positions, *n* (%)	40 (63.5)	27 (65.9)	0.142	0.095; [–0.040 – 0.230]
Speakers with multiple degrees, *n* (%)	24 (38.1)	14 (34.2)	0.161	0.114; [–0.056 – 0.286]
MD as a higher degree, *n* (%)	40 (63.5)	25 (61.0)	0.060	3.00; [–0.166 – 6.166]
PhD as a higher degree, *n* (%)	8 (12.7)	9 (22.0)	0.828	–0.031; [–0.351 – 0.289]
Medical students, *n* (%)	11 (17.5)	5 (12.2)	0.389	0.078; [–0.119 – 0.276]
Speakers who have previously spoken at conferences, *n* (%)	49 (77.8)	28 (68.3)	0.160	4.20; [–2.566 – 10.966]
Citation median [IQR]	253 [45–753]	53 [0–410]	0.342*	—

*Note*: Table 2 presents an overall comparison of speaker credentials between high-income countries (HICs) and low and middle-income countries (LMICs). β is the coefficient of the linear regression analysis; 95% CI is the confidence interval.

*The *p*-value of citation median was generated from a Mann–Whitney *U* test.

### Speakers’ specialty

The speakers’ medical specialties that had an overall higher frequency were obstetrics and gynecology with 10.6% (*n* = 11/104) of speakers, followed by global surgery (*n* = 8/104, 7.7%), neurosurgery (*n* = 8/104, 7.7%), trauma surgery (*n* = 7/104, 6.7%), pediatric surgery (*n* = 6/104, 5.8%), general surgery (*n* = 6/104, 5.8%), and public health specialists (*n* = 6/104, 5.8%). The specialties with the lowest frequencies were anesthesiology, transplant surgery, otorhinolaryngology, and pediatrics, each representing 0.9% (*n* = 1/104). These were followed by global health (*n* = 2/104, 1.9%), surgical oncology (*n* = 2/104, 1.9%), urology surgery (*n* = 2/104, 1.9%), bariatric surgery (*n* = 2/104, 1.9%), vascular surgery (*n* = 2/104, 1.9%), orthopedic surgery (*n* = 3/104, 2.9%), internal medicine *n* = 3/104, 2.9%), thoracic surgery (*n* = 4/104, 3.8%), plastic and reconstructive surgery (*n* = 5/104, 4.8%), and endocrine surgery (*n* = 5/104, 4.8%) (Table 3).

**Table 3:** Speakers’ professional specialties.

SPEAKERS’ PROFESSIONAL SPECIALTY, *N* (%)	2020 (*N* = 22)	2021 (*N* = 19)	2023 (*N* = 21)	2024 (*N* = 24)	2025 (*N* = 18)	TOTAL (*N* = 104)
Obstetrics and gynecology	1 (4.5)	1 (5.3)	3 (14.3)	3 (12.5)	3 (16.7)	**11 (10.6)**
Global surgery	2 (9.1)	0 (0)	2 (9.5)	1 (4.2)	3 (16.7)	**8 (7.7)**
Neurosurgery	2 (9.1)	2 (10.5)	1 (4.7)	1 (4.2)	2 (11.1)	**8 (7.7)**
Trauma surgery	2 (9.1)	3 (15.8)	1 (4.7)	0 (0)	1 (5.6)	**7 (6.7)**
Pediatric surgery	0 (0)	3 (15.8)	0 (0)	3 (12.5)	0 (0)	**6 (5.8)**
General surgery	0 (0)	2 (10.5)	2 (9.5)	2 (8.3)	0 (0)	**6 (5.8)**
Public health	0 (0)	0 (0)	3 (14.3)	2 (8.3)	1 (5.6)	**6 (5.8)**
Endocrine surgery	0 (0)	2 (10.5)	1 (4.7)	2 (8.3)	0 (0)	**5 (4.8)**
Plastic and reconstructive surgery	1 (4.5)	1 (5.3)	0 (0)	2 (8.3)	1 (5.6)	**5 (4.8)**
Thoracic surgery	1 (4.5)	2 (10.5)	0 (0)	0 (0)	1 (5.6)	**4 (3.8)**
Internal medicine	1 (4.5)	2 (10.5)	0 (0)	0 (0)	0 (0)	**3 (2.9)**
Orthopedic surgery	1 (4.5)	0 (0)	0 (0)	2 (8.3)	0 (0)	**3 (2.9)**
Global health	0 (0)	0 (0)	2 (9.5)	0 (0)	0 (0)	**2 (1.9)**
Surgical oncology	0 (0)	0 (0)	0 (0)	2 (8.3)	0 (0)	**2 (1.9)**
Urology surgery	0 (0)	0 (0)	1 (4.7)	1 (4.2)	0 (0)	**2 (1.9)**
Bariatric surgery	0 (0)	0 (0)	0 (0)	0 (0)	2 (11.1)	**2 (1.9)**
Vascular surgery	1 (4.5)	0 (0)	0 (0)	0 (0)	1 (5.6)	**2 (1.9)**
Anesthesiology	0 (0)	1 (5.3)	0 (0)	0 (0)	0 (0)	**1 (0.9)**
Otorhinolaryngology	0 (0)	0 (0)	0 (0)	1 (4.2)	0 (0)	**1 (0.9)**
Pediatrics	0 (0)	0 (0)	0 (0)	1 (4.2)	0 (0)	**1 (0.9)**
Transplant surgery	0 (0)	0 (0)	1 (4.7)	0 (0)	0 (0)	**1 (0.9)**

*Note*: Table 3 outlines the distribution of speaker professional specialities across the GEIGS General Assemblies from 2020 to 2025.

### General assembly session topics

A total of 56 sessions were held throughout the five editions of the GEIGS GA. The GA edition of 2023 had the highest number of sessions (*n* = 13/56, 23.2%), followed by 2024 and 2025 each representing 21.4% (*n* = 12/56). The 2021 GA edition had the fewest number of sessions (*n* = 9/56, 16.1%), followed by 2020 (*n* = 10/56, 17.9%). We grouped the session topics into seven main themes relevant to global surgery and gender equity. Overall, the most frequently discussed theme was *Diversity, Gender Equity, and Inclusion* (*n* = 17/56, 30.4%), followed by *Advocacy, Policy, and Structural Change* (*n* = 14/56, 25.0%), *Research and Innovation* (*n* = 7/56, 12.5%), *Mentorship and Career Development* (*n* = 6/56, 10.7%), *Work-Life Balance and Gender Expectations* (*n* = 4/56, 7.1%), *GEIGS Internal Insights* (*n* = 4/56, 7.1%), and *Regional Insights* (*n* = 3/56, 5.4%). The specific session titles and yearly counts are described in Table 4.

**Table 4:** GA sessions and main themes.

	2020 (*N* = 10)	2021 (*N* = 9)	2023 (*N* = 13)	2024 (*N* = 12)	2025 (*N* = 12)	TOTAL (*N* = 56)
**Main Themes**	**Session Title**					
**Diversity, Gender Equity, and Inclusion, *n* (%)**	**2 (20.0)**	**5 (55.6)**	**2 (15.4)**	**5 (41.7)**	**3 (25.0)**	**17 (30.4)**
	Intersectionality	Patient Preference on Their Physician Gender? How to Overcome It	The Urgent Need for Gender Affirming Care	Advancing Transgender Health Equity: A Call to Action	Education Without Borders: Collaborative Efforts for Inclusive and Quality Learning	
	Diversity, Equity, and Justice in Global Surgery	Male Allies—How Can Majority Identities Take Steps to Further Gender Equity	Women in Global Surgery Equity in Leadership	Advancing Gender Equity in Global Surgical Education	Gender Equity in Surgical Training and Workforce Development	
		Panel with Global Women Surgeon		Global Access to Gender Affirming Surgery and Transgender Care	From Representation to Transformation: Lived Experiences and Leadership in the Fight Against Inequality	
		Intersectionality		Ensuring Gender Inclusive and Safe Spaces in Global Health Conferences		
		Gender Inequities in COVID-19 Leadership and How We May All “Build Back Better”		How Not to Make Diversity Equity and Inclusion a Checkbox Exercise		
**Advocacy, Policy, and Structural Change, *n* (%)**	**1 (10.0)**	**2 (22.2)**	**2 (15.4)**	**4 (33.3)**	**5 (41.7)**	**14 (25.0)**
	Advocacy Session	Global Surgery the Current Landscape	Advocacy for Global Surgery Insights	How to Minimize Duplicating Efforts by Global Surgery Organizations	Empowerment Access and Sustainable Well-Being	
		Advocacy Session	Insights on Climate Change and Global Health	Role of Gender in Countryside Surgical, Anesthesia, and Obstetric Plans and Policies	Advocacy in Gender Equity: Using Social Media to Build Community and Drive Change	
				Workplace Policies Guarantee Gender Equality in the Surgical Workforce	Saving Mothers, Saving Futures: Blood, Health, and Human Rights	
				Empowering Change Through Allyship	Women Leading Climate Action in Surgery: A Path Toward SDG 13	
					Building Equitable Surgical Systems: The Role of Peace, Justice, and Gender Inclusive Institutions	
**Research and Innovation, *n* (%)**	**1 (10.0)**	**1 (11.1)**	**3 (23.1)**	**1 (8.3)**	**1 (8.3)**	**7 (12.5)**
	Research Session	Research Session	Journal Club as a Mean to Advance Research	Representation of Women in Scientific Research Journals	Applications of AI in Global Surgery	
			Innovations in Global Surgery			
			Breaking Barriers and Promoting Equity in Research in Surgery			
**Mentorship and Career Development, *n* (%)**	**1 (10.0)**	**1 (11.1)**	**2 (15.4)**	**1 (8.3)**	**1 (8.3)**	**6 (10.7)**
	Mentorship Session	Is an Academic Career the Right Fit for Me?	Mentorship: How to Make the Best of This Relationship	Navigating Mentor–Mentee Relationships	Identifying Microaggressions in Surgical Systems and Learning How to Deal with Them	
			Youth in Global Surgery			
**Work-Life Balance and Gender Expectations, *n* (%)**	**0 (0)**	**0 (0)**	**3 (23.1)**	**1 (8.3)**	**0(0)**	**4 (7.1)**
			Women in Surgery: The Experience of The First Woman to Perform a Liver Transplant	Escaping War and Conflict as a Female Pediatric Surgeon in Afghanistan		
			Family Planning and Work-Life Integration			
			Addressing Health-Care Workers’ Mental Health			
**Regional Insights, *n* (%)**	**3 (30.0)**	**0 (0)**	**0 (0)**	**0 (0)**	**0(0)**	**3 (5.4)**
	Centering the Global South					
	Regional Session (WPRO and SEARO)					
	Regional Session (EURO and AFRO)					
**GEIGS Internal Insights, *n* (%)**	**2 (20.0)**	**0 (0)**	**1 (7.6)**	**0 (0)**	**1(8.3)**	**4 (7.1)**
	Panel with Junior Advisors and Founders		History of GEIGS		History of GEIGS	
	Message from the Chairs					

*Note*: Table 4 describes the main themes and corresponding session titles featured at GEIGS General Assemblies from 2020 to 2025. WPRO: Western Pacific Region; SEARO: Southeast Asia Region; AFRO: African Region; EURO: European Region; SDGs: Sustainable Development Goals.

## Discussion

This retrospective analysis of speaker representation at the GEIGS GA from 2020 to 2025 provides a descriptive case study, demonstrating consistently high female participation while also highlighting persistent gaps in research visibility and specialty representation. Across all assemblies, women comprised 83.7% of speakers, a proportion markedly higher than global trends, where women often constitute fewer than 30% of academic faculty in HICs and even fewer in LMICs [11, 12]. These findings illustrate that a deliberate, mission-driven approach to conference design, emphasizing equitable participation, can support female engagement and visibility within the global surgery community [13, 14].

LMIC speakers accounted for 39.4% of participants, many of whom held leadership roles or advanced degrees, consistent with the conference’s emphasis on diverse involvement. A high proportion of speakers had prior conference experience (68.3%), and early-career professionals were represented in both LMIC and HIC groups (12.2% vs. 17.5% medical students), suggesting that the assembly included a broad range of career stages. LMIC speakers had a lower median citation count (53 vs. 253), which may reflect broader disparities in research infrastructure, access to mentorship, and publishing opportunities [15–17]. Leadership roles and multiple-degree attainment were broadly comparable between LMIC and HIC speakers, showing that diverse participation is achievable in this conference context [7].

Distribution across specialties was highest in obstetrics and gynecology, neurosurgery, global surgery, and trauma surgery, with underrepresentation in fields such as transplant, urology, and anesthesiology. These patterns may be influenced by existing gender imbalances within specialties and differences in access to global health opportunities [18–20]. Future assemblies may consider targeted strategies to balance specialty representation, track speaker trajectories over time, and implement inclusive recruitment practices to enhance diversity across disciplines [21, 22].

The most frequently addressed topic across the five assemblies was Diversity, Gender Equity, and Inclusion, highlighting the central role of these principles within GEIGS and the broader global surgery academic community. Sessions on Advocacy, Policy, and Structural Change and Research and Innovation reflected the organization’s focus on academic advancement. Mentorship, Career Development, and Work-Life Balance sessions emphasized personal and professional sustainability, while sessions on Regional and GEIGS Internal Insights demonstrate the breadth of topics covered. Collectively, these sessions illustrate a multifaceted approach to promoting representation in global surgery and the inclusion of diverse perspectives.

This study has several limitations. The small sample size limits the generalizability of our findings, and the results should be interpreted as primarily descriptive. The high female representation likely reflects GEIGS’s mission-driven focus on gender inclusivity rather than broader systemic change. Selection bias is inherent, as a higher proportion of women and speakers from underrepresented backgrounds may have been included compared to other global surgical conferences. Despite these limitations, these data provide insight into how conference planning can shape speaker composition within a single global surgery forum.

In conclusion, intentional planning is key to promoting the participation of women and individuals from underrepresented groups in academic and professional speaking engagements. The GEIGS experience shows that deliberate, mission-driven program design can achieve high female participation and meaningful representation of LMIC professionals. While these findings may not be generalizable to all contexts, the mentorship programs and policies to support underrepresented voices employed by GEIGS provide a potential framework for other organizations seeking to enhance inclusivity.

### Lessons learned

- Deliberate design and leadership may enhance women’s and LMIC participation in global surgery events.
- Engaging and mentoring LMIC and underrepresented speakers can reduce structural barriers and broaden specialty participation.
- Monitoring demographics and session topics can track progress toward inclusion goals.
- Collaborative networks and mentorship programs can empower early-career professionals and amplify underrepresented voices.
